# Foundational Statistical Principles in Medical Research: A Tutorial on Odds Ratios, Relative Risk, Absolute Risk, and Number Needed to Treat

**DOI:** 10.3390/ijerph18115669

**Published:** 2021-05-25

**Authors:** Thomas F. Monaghan, Syed N. Rahman, Christina W. Agudelo, Alan J. Wein, Jason M. Lazar, Karel Everaert, Roger R. Dmochowski

**Affiliations:** 1 Department of Urology, University of Texas Southwestern Medical Center, Dallas, TX 75390, USA; 2 Department of Urology, Yale University School of Medicine, New Haven, CT 06520, USA; syed.rahman@downstate.edu; 3 Division of Cardiovascular Medicine, Department of Medicine, SUNY Downstate Health Sciences University, Brooklyn, NY 11203, USA; christina.agudelo@downstate.edu (C.W.A.); Jason.lazar@downstate.edu (J.M.L.); 4 Division of Urology, Perelman School of Medicine at the University of Pennsylvania, Philadelphia, PA 19104, USA; alan.wein@pennmedicine.upenn.edu; 5 Department of Human Structure and Repair, Ghent University, 9000 Ghent, Belgium; karel.everaert@uzgent.be; 6 Department of Urological Surgery, Vanderbilt University Medical Center, Nashville, TN 37232, USA; roger.dmochowski@vumc.org

**Keywords:** basics, biostatistics, fundamentals, introduction, methodology, odds, ratio, relative, risk, statistics

## Abstract

Evidence-based medicine is predicated on the integration of best available research evidence with clinical expertise and patient values to inform care. In medical research, several distinct measures are commonly used to describe the associations between variables, and a sound understanding of these pervasive measures is foundational in the clinician’s ability to interpret, synthesize, and apply available evidence from the medical literature. Accordingly, this article aims to provide an educational tutorial/topic primer on some of the most ubiquitous measures of association and risk quantification in medical research, including odds ratios, relative risk, absolute risk, and number needed to treat, using several real-world examples from the medical literature.

## 1. Introduction

Evidence-based medicine is predicated on the integration of best available research evidence with clinical expertise and patient values to inform care [1]. Despite increasing acceptance of the concept of evidence-based practice [2,3], there paradoxically exists a growing body of research suggesting a major divide between current medical knowledge and practice [4]. Namely, there exists a major lag from the publication of evidence to its translation into clinical practice [5], such that the use of current best evidence remains suboptimal, directly contributing to preventable harm, poor patient outcomes, and wasted resources [6].

One well-described contributory factor underlying the gap between current evidence and practice is inconsistent statistical literacy among clinicians [4]. Statistical literacy, which is generally viewed as a prerequisite for medical research interpretation and synthesis, is thus considered a core tenet of evidence-based best practice [7,8,9]. Although the vast majority of physicians indeed consider statistics to be important to their work [10], several prior studies have reported a poor understanding of basic statistical principles among clinicians spanning multiple medical specialties [11,12,13,14]. Perhaps the most convincing evidence of suboptimal statistical literacy among clinicians stems from a well-conducted multi-national randomized survey from Johnston et al., which investigated clinician understanding of different statistical formats for presenting continuous outcomes from meta-analyses, and found that all assessed measures of treatment effect (i.e., measures of the strength of the relationship between two variables) were poorly understood or perceived to have limited usefulness by a majority of respondents [15].

Notably, beyond clinicians and other consumers of medical research, shortcomings in the application and interpretation of statistics among producers of medical research have also been extensively described. Numerous studies have been published on the quality of statistical reporting in biomedical literature, which consistently find large proportions of included articles to contain errors in the application, analysis, or interpretation of statistics—errors often serious enough to challenge the validity of the authors’ conclusions [16]. Importantly, while statistical errors can indeed be found in more advanced statistical methods, many errors are in basic, rather than complex, statistical concepts [17].

One pragmatic and increasingly popular solution for improving statistical knowledge within the medical community is the use of more accessible language, particularly in the context of core statistical principles, as to make these concepts more comprehensible [4,18]. In view of the well-described shortcomings in statistical interpretation in the medical community, particularly with respect to measures of treatment effect, the present article aims to provide a comprehensive-yet-comprehensible tutorial on commonly reported measures of association and risk quantification in the medical literature, including odds ratios, relative risk, absolute risk, and number needed to treat, using several examples from real-world research publications.

## 2. Measures of Disease Frequency

Understanding common terminology related to disease frequency, including the distinction between prevalence and incidence, is a key starting point in the discussion of measures of association in the medical literature. **Prevalence** denotes the proportion of all people in the study population who have the outcome of interest (e.g., disease or condition) at a particular time [19].

$$Prevalence=\frac{\#\;People\;Affected}{\#\;People\;in\;Study\;Population}.$$

Suppose that we are trying to establish the baseline clinical characteristics of 10,000 middle-aged adults enrolled in a 5-year study on the effects of a new wonder drug, ‘utsouthwesternide’, for stroke prevention. We find that 1000 out of 10,000 enrolled subjects have diabetes at the beginning of the study. In this scenario, the baseline prevalence of diabetes is 1000/10,000, or 100 cases per 1000 people in the study population. 

Note that researchers may also distinguish between **point prevalence** and **period prevalence**, with the former referring to the prevalence of a given outcome at a point in time, and the latter referring to the prevalence during a period of time, which is similar to point prevalence except that the ‘point’ is broader (e.g., multiple years). For example, we may report a baseline ‘period prevalence’ if it had taken us 3 years to enroll and examine all 10,000 people who participated in the above-mentioned ‘utsouthwesternide’ trial. 

That said, it is rare for researchers to be able to determine disease status in the entire study population at exactly the same moment in time, and there is no strict temporal cutoff by which to differentiate a ‘point’ from a ‘period’. Accordingly, while it is important to be familiar with these specifiers for prevalence, point prevalence and period prevalence may often be difficult to delineate in practice. Another related term is **lifetime prevalence**, which denotes the proportion of individuals in a population that experience the outcome of interest at any time in their life [20].

**Incidence**, in contrast to prevalence, is the proportion of at-risk subjects who develop the outcome of interest [21].

$$Incidence=\frac{\#\;New\;Cases}{\#\;At-Risk\;People\;in\;Study\;Population}.$$

As subjects known to have diabetes are, by definition, no longer ‘at risk’ for the disease, only 9000/10,000 subjects in the ‘utsouthwesternide’ study can be considered at risk for diabetes at the start of the trial.

Incidence can be expressed either as a proportion or a rate. The **incidence proportion**, or **cumulative incidence**, is the probability that an outcome has occurred before a given time among all subjects at risk for the disease [21]. The **incidence rate** is the frequency at which an event occurs over a specified time period, often expressed in ‘person-time’ [21]. **Person-years** denotes the cumulative amount of time (in years) that all at-risk subjects are observed for the outcome of interest. For example, if 9000 at-risk subjects are each observed for 5 years, the total person-years of observation is 45,000. (Subjects who develop the outcome of interest in the middle of the study, or who fail to complete the study (e.g., dropout, loss to follow-up, or death), only contribute to ‘years of observation’ until the moment any such event occurs).

Now suppose that we are able to reexamine all participants at the end of the 5-year ‘utsouthwesternide’ trial (and that, miraculously, no subjects were diagnosed with diabetes in the interim, and all enrolled subjects completed the study). We now identify 500 new cases of diabetes from the subset of participants who did not have the disease at baseline (*n* = 9000). In this scenario, the cumulative incidence of diabetes during the study period is 500/9000, or 55.5 cases per 1000 subjects (i.e., 5.6%) over five years. Alternatively, we may find it more meaningful to report the incidence rate of diabetes, which is equal to 500 new cases/45,000 person-years, or 11.1 cases per 1000 person-years.

## 3. Quantifying Risk

### 3.1. Risk Versus Odds

In general conversation, the terms ‘risk’ and ‘odds’ are often used interchangeably to refer to the ‘chance’ of something happening. However, the usage of these terms in this manner is problematic in the context of medical research, where ‘risk’ and ‘odds’ have very specific connotations and must be carefully distinguished from one another. Risk, a relatively more familiar concept for most physicians, denotes the probability that an outcome will occur. Odds, in contrast, denotes the probability that an event occurs divided by the probability that the event does not occur.

The subtle distinction between ‘risk’ and ‘odds’ is best understood using a real example from the medical literature. In a 4-year, randomized, placebo-controlled, blinded parallel-group study, Andriole et al. sought to determine the effect of dutasteride (a 5α-reductase inhibitor) on the incidence of prostate cancer in men 50–75 years of age [22]. The primary study results are summarized in Table 1. 

In this study, the overall risk of prostate cancer was equal to 1517/6729, or 0.23 (number of prostate cancer cases/(all outcomes, i.e., prostate cancer + no prostate cancer)). Following this equation, we can also report that the risk of prostate cancer was equal to 659/3305, or 0.20, in the dutasteride group and 858/3424, or 0.25, in the control group. While dutasteride and prostate cancer constitute the intervention and outcome of interest, respectively, in this particular study, the concept of risk can be applied more broadly following a standard ‘contingency table’, wherein risk for the outcome of interest is expressed as (A/(A + B)) in the intervention group and (C/(C + D)) in the control group (Table 2). 

Using these same data, the overall odds of prostate cancer were 1517/5219, or 0.29 (number of prostate cancer cases/(number of non-cases, i.e., no prostate cancer)). Following this equation, the odds of prostate cancer were 659/2646, or 0.25, in the dutasteride group and 858/2566, or 0.33, in the control group. Following our standard contingency table, the odds of the outcome of interest (e.g., prostate cancer) can be expressed as (A/B) in the intervention (e.g., dutasteride pharmacotherapy) group and (C/D) in the control group (Table 2).

Note that, for a given dataset, the ‘chance’ of death appreciably differs when expressed as risk (0.23) versus odds (0.29). Importantly, the magnitude of divergence between risk and odds actually varies based on the event rate. As illustrated in Table 3, if prostate cancer were even more frequent than reported in the present study (*n* = 1517 cases), the difference between the risk of prostate cancer and the corresponding odds of prostate cancer would be even greater in magnitude, wherein the odds would further appear to ‘overestimate’ the risk of prostate cancer if the values computed for the risk and odds were to be mistakenly interpreted as interchangeable. Conversely, as the frequency of the outcome decreases, the odds of prostate cancer more closely align with the risk of prostate cancer.

### 3.2. Relative Risk and Odds Ratios

In Section 3.1, we applied our understanding of risk and odds by calculating these quantities separately in the intervention (dutasteride) group and control group from the study by Andriole et al. [22]. While these are important fundamental concepts, what, in and of itself, does a ‘risk of 0.20’, or ‘odds of 0.25’, in the treatment group really mean? It stands to reason that a ‘risk of 0.20’ in the treatment group would have very different connotations if the risk in the control group was 0.25 than it would if the risk in the control group was 0.15. 

In other words, for a prostate cancer risk of 0.20 in the treatment group versus 0.25 in the control group, as was the case in the present study, we can conclude that dutasteride reduced the risk of prostate cancer. Conversely, if the risk of prostate cancer were to remain 0.20 in the treatment group, but hypothetically were found to be 0.15 in the control group, we would conclude the opposite—that dutasteride increased the risk of prostate cancer.

**Relative risk** (encompassing both **risk ratios** and **rate ratios**), as well as **odds ratios**, are ubiquitous in the medical literature because they allow for meaningful assessment of the relationship between an intervention/exposure and an outcome by comparing risk and odds, respectively, between groups. Relative risk and odds ratios are simply the risk or odds, respectively, of an outcome in one group divided by the risk or odds of the outcome in another other group [23]. For example, recall that, following our standard contingency table (Table 2), the risk of a positive outcome among study subjects who have had the exposure/intervention is equal to (A/(A + B)), and the risk of a positive outcome among non-exposed/control subjects is equal to (C/(C + D)). Thus, the relative risk (i.e., risk in one group ‘relative’ to risk in the other group) can be expressed as follows:

$$Relative\;Risk=\frac{Risk\;of\;Disease\;in\;Exposure\;or\;Intervention\;Group}{Risk\;of\;Disease\;in\;the\;Nonexposed\;or\;Control\;Group}=\frac{\frac{A}{\left(A+B\right)}}{\frac{C}{\left(C+D\right)}}=\frac{A/\left(A+B\right)}{C/\left(C+D\right)}.$$

Relative risk and odds ratios are interpreted similarly, in that a relative risk (or odds ratio) of 1.0 indicates no difference in risk (or odds) between groups; a relative risk (or odds ratio) >1.0 indicates an increased risk (or odds) among exposed/intervention versus non-exposed/control groups; and a relative risk (or odds ratio) <1.0 indicates a decreased risk (or odds) among exposed/intervention versus non-exposed/control groups.

Returning to the dutasteride study, recall that the risk of prostate cancer was 0.20 in the intervention group (A/(A + B)) and 0.25 in the control group (C/(C + D)), corresponding to a relative risk, or **risk ratio**, of 0.20/0.25, or 0.80. In other words, we can conclude that subjects who received dutasteride had 0.80 times the risk of prostate cancer compared to subjects who did not receive dutasteride. 

The present study results can alternatively be interpreted using the **percent relative effect**. The percent relative effect, as the name suggests, denotes the relative change in risk of an event in the exposed/intervention group compared to the non-exposed/control group. When the relative risk is less than 1, as was the case in the present study (relative risk = 0.80), the percent relative effect, or **relative risk reduction**, is equal to ((1 − relative risk) × 100) (e.g., (1 − 0.80) × 100 = 20% decrease in risk). A 20% decrease in risk can be understood to mean that those subjects who took dutasteride had a 20% decrease in risk of prostate cancer compared to those who did not take dutasteride. For scenarios where the relative risk is greater than 1, the percent relative effect, or **relative risk increase**, is equal to ((relative risk − 1) × 100) (e.g., for a relative risk of 5.3, ((5.3 − 1) × 100) = 430% increase in risk). A 430% increase in risk indicates that those subjects who had the intervention/exposure had a 430% increase in risk above, or a 530% greater risk, than the control group. **Preventable fraction among the exposed** and **attributable fraction among the exposed** are sometimes used synonymously with relative risk reduction and relative risk increase, respectively [24].

Relative risk is a general term that encompasses both risk ratio (exemplified above) and rate ratio. Let us approach the concept of rate ratio using another real-world example from a cohort study—a study design for which relative risk is also frequently assessed. Recall that, in a cohort study, a disease-free study sample is stratified based on the presence or absence of an exposure and then analyzed (either prospectively or retrospectively) to compare disease frequency in the exposed versus non-exposed group [25]. 

In this context, relative risk broadly quantifies the relationship between the risk of the outcome in the exposed group and the risk of the outcome in the non-exposed group. Thus, the risk ratio would be the cumulative incidence of the outcome in the exposed group divided by the cumulative incidence of the outcome in the non-exposed group. A **rate ratio** is analogous to the risk ratio except that it instead compares the incidence rate, rather than the cumulative incidence, between groups [26].

In a population-based retrospective cohort study of more than 2.5 million older adults in Ontario, Canada, Wallis et al. sought to characterize the association between antithrombotic medication use and complications related to hematuria, defined as an emergency department visit, hospitalization, or urologic procedure to evaluate or manage gross hematuria [27]. A total of 808,897 subjects received at least one antithrombotic prescription over the study period, and 1,709,167 subjects received no antithrombotic agents. During a median follow-up period of 7.3 years, hematuria-related complications occurred at a rate of 123.95 events per 1000 person-years among exposed subjects and 80.17 events per 1000 person-years among non-exposed subjects. Based on these incidence rates, the rate ratio can be estimated to be (123.95/1000)/(80.17/1000) or 1.55 (unadjusted for simplicity).

As demonstrated by the practical examples that we have considered to this point, relative risk is frequently computed in interventional trials and cohort studies comparing the cumulative incidence (i.e., risk ratio) or incidence rates (i.e., rate ratio) between groups. An **odds ratio**, defined as the ratio of odds of an event in one group versus the odds of an event in the other group, can analogously be derived for interventional trials and cohort studies. However, in studies comparing the incidence of an event (e.g., clinical trials and cohort studies), relative risk is often the preferred measure of association because the odds ratio will always ‘overstate’ the magnitude of the effect (i.e., the odds ratios will be smaller than the relative risk for risk ratios less than 1 and larger than the relative risk for risk ratios greater than 1). [28]. Notably, as was the case for ‘risk’ versus ‘odds’ (Table 3), an increasingly pronounced divergence can be observed between the relative risk and corresponding odds ratios as the event rate increases (Table 4).

In contrast to clinical trials and cohort studies, odds ratios are not only preferred in case-control studies, but are often the only measure of association that can be applied to this type of study design. Recall that case-control studies are observational studies wherein the investigators first identify cases (i.e., subjects known to have the outcome of interest) and controls (i.e., subjects known to be free of the outcome), and then compare the frequency of exposures between groups to identify potential contributory factors [29]. 

By definition, the ratio of cases to controls is usually determined by study investigators, such that the proportion of cases in the study sample does not reflect the actual risk of disease in the population. Although this study design inherently precludes meaningful assessment of relative risk, odds ratios can still be readily applied to case-control studies, and are used to denote the odds of exposure among cases versus controls [30]. (As may be surmised from Table 4, the ‘rare disease assumption’ posits that, when a disease outcome of interest is exceedingly uncommon, the odds ratio of exposure in case-control studies can be used to estimate the relative risk [31]; however, this technique remains controversial [32]). 

Let us now apply the concept of odds ratios to an actual case-control study from the medical literature. Lorenzo-González et al. sought to assess the association between lung cancer and residential radon exposure in Northwestern Spain among adults with no smoking history [33]. Radon exposure data, as ascertained from radon detectors provided to participants, were obtained from 489 subjects with biopsy-confirmed primary lung cancer and 751 controls of similar sex and age. Analysis of participant characteristics revealed high residential radon exposure (≥200 Becquerel (Bq)/m^3^) in 192/489 (39.3%) cases compared to 195/751 (26.0%) controls (Table 5). Correspondingly, we can estimate the odds ratio of high radon exposure in cases versus controls to be 1.84 ((192/297)/(195/556)).

Based on these data, we can conclude that individuals with lung cancer were 1.84 times more likely to have a history of high residential radon exposure compared with those without lung cancer. (Note that it is not appropriate to interpret this odds ratio as saying ‘individuals who have a history of high residential radon exposure are 2.95-times more likely to develop lung cancer than those who do not have a history of high residential radon exposure’. The reason for this important distinction is that a case-control study starts from the outcome of interest and only then examines exposure status).

### 3.3. Risk Difference and Rate Difference

Relative risk and similar terms such as percent relative effect, as well as odds ratios, are all considered relative measures of association because they convey the risk (or odds) in one group ‘relative’ to the risk (or odds) in another group. However, the current CONSORT (Consolidated Standards of Reporting Trials) guidelines recommend that both relative and non-relative (i.e., ‘absolute’) associations be reported [34]. 

Such duplicate reporting is necessitated, in large part, by the fact that relative measures of association have the potential to cause readers to overestimate the efficacy of an intervention [35]. To understand this, recall that, using data from the dutasteride study, we computed a relative risk reduction of 20% based on a prostate cancer risk of 0.20 in the intervention group and 0.25 in the control group ((1 − 0.20/0.25) × 100 = 20%).

Importantly, a relative risk reduction of 20%, in and of itself, may just as well have been derived from a risk ratio of 0.64/0.80 ((1 − 0.64/0.80) × 100 = 20%) or, alternatively, a risk ratio of 0.04/0.05 ((1 − 0.04/0.05) × 100 = 20%). In the case of 0.04 versus 0.05, it becomes apparent how easily relative risk reduction could be selectively reported in order to suggest meaningful benefit from an intervention despite modest absolute differences between the intervention group risk versus control group risk. 

**Risk difference**, as the name suggests, is simply the difference in the risk for an outcome between study groups. The risk difference can be derived for interventional studies as well as for observational cohort studies following the same general formula (Table 2):

$$Risk\;Difference=\frac{A}{\left(A+B\right)}-\frac{C}{\left(C+D\right)}.$$

**Rate difference** is analogous to risk difference except for the fact that the difference is expressed in units of time (i.e., incidence rate rather than cumulative incidence). In other words, the rate difference denotes the number of exposed subjects with the condition divided by the person-years of observation in that group, minus the number of non-exposed subjects with the condition divided by the person-years of observation in that group.

In a retrospective cohort study, Fang et al. explored the association between bladder diverticula and bladder cancer risk in a Taiwanese population-based cohort of 10,662 hospitalized urology patients [36]. Bladder cancer was identified in 37/2134 (1.7%) subjects with bladder diverticula and 58/8528 subjects without bladder diverticula (0.7%) (Table 6). For this cohort study example, the risk difference was equal to 37/2134 − 58/8528, or 0.0105 (1.05%). The authors also reported that subjects with bladder diverticula were observed for a total of 11,674 person-years, and subjects without bladder diverticula were observed for a total of 47,711 person-years, corresponding to bladder cancer incidence rates of 3.17 per 1000 person-years and 1.22 per 1000 person-years, respectively. Thus, the rate difference between groups is equal to 1.95 cases per 1000 person-years.

Many frequently confused terms used to describe associations in clinical trials are fundamentally based on the risk difference. **Absolute risk increase**, computed as the risk in exposed/intervention subjects minus the risk in non-exposed/control subjects, can be used to specify risk difference in scenarios when the risk of an outcome is increased by the exposure or intervention. **Absolute risk reduction**, computed as the risk in non-exposed/control subjects minus the risk in exposed/intervention subjects, can be used to specify risk difference when the risk of an outcome is decreased by the exposure/intervention. An alternative term for risk difference is **attributable risk** (i.e., excess risk that can be attributed to having had the exposure); however, this term may erroneously imply a cause-and-effect relationship between the exposure and the outcome in observational studies.

### 3.4. Number Needed to Treat and Number Needed to Harm

The inverse of the rate difference or risk difference is known either as the **number needed to treat** or **number needed to harm**, depending on whether the exposure/intervention decreases or increases the risk of the outcome, respectively [37]. As cohort studies do not involve an intervention, some authors instead use **number needed to be exposed** to apply this concept, but the basic premise remains the same [38].

The number needed to treat is a particularly effective measure for communicating the effectiveness of an intervention in absolute terms because it signifies the average number of participants who would need the exposure/intervention to prevent one additional poor outcome. For example, the reported rate difference (i.e., absolute risk reduction) of 5.1% in the dutasteride trial corresponds to a number needed to treat of 20. In other words, 20 subjects need to be treated with dutasteride to prevent one additional case of prostate cancer within 4 years (study duration). 

By the same token, the number needed to harm quantifies the number of persons who would need to be exposed to a risk factor over a specific period to cause one additional poor outcome. For example, in the cohort study from Fang et al., our calculated risk difference of 0.0105 corresponds to a number needed to harm (i.e., ‘number needed to be exposed for one person to be harmed’) of 95. Alternatively, in view of the observed rate difference of 1.95 cases/1000 person-years, we can conclude that subjects with bladder diverticula had one additional case of bladder cancer per 513 person-years compared to subjects without bladder diverticula. As may be surmised from these examples, a higher number needed to treat indicates that a treatment is less effective while a lower number needed to harm would be expected for more deleterious interventions and exposures.

## 4. Common Pitfalls

1. In quantifying disease frequency, the denominator for prevalence is equal to the total number of subjects in a study population, whereas the denominator for incidence includes only at-risk subjects, such that subjects who already have the disease would be counted in the denominator for prevalence but not for incidence [39].

2. While ‘risk’ and ‘odds’ are often used interchangeably in general conversation, they take on very particular meanings in medical research. Risk is the probability that an outcome will occur, expressed as the number of positive outcomes divided by the total number of outcomes, and odds denotes the probability that an event will occur divided by the probability that an event will not occur [40].

3. Odds ratios will always ‘overstate’ the magnitude of an effect (i.e., odds ratios will be smaller than relative risk for risk ratios less than 1 and larger than relative risk for risk ratios greater than 1) [40].

4. Relative risk cannot be assessed in most case-control studies because the proportion of cases in case-control study samples is usually determined by investigators, such that the proportion of cases does not reflect the actual risk of disease in the population [41].

5. Readers should be wary of studies that publish only relative measures of association, which, in and of themselves, can be manipulated to suggest meaningful benefit from an intervention despite modest absolute differences in risk between groups, causing readers to overestimate the efficacy of an intervention [42].

## 5. Conclusions

Prevalence denotes the proportion of all subjects who have the outcome of interest at a particular time, and incidence signifies the proportion of at-risk subjects who develop the outcome of interest, expressed either as a cumulative proportion (i.e., before a given time) or rate. ‘Risk’ is the probability that an outcome will occur, expressed as the number of positive outcomes divided by the total number of outcomes, and ‘odds’ denote the probability that an event will occur divided the probability that an event will not occur. Relative risk and odds ratios are simply the risk or odds, respectively, of an outcome in one group divided by the risk or odds of the outcome in another group. 

Relative risk is often preferred over odds ratios for quantifying risk in clinical trials and observational cohort studies, but cannot be ascertained in most case-control studies, wherein odds ratios are frequently assessed instead. Relative risk may be reported as the ‘percent relative effect’, equal to ((1 − relative risk) × 100) when the relative risk is less than 1 (i.e., ‘relative risk reduction’), and ((relative risk − 1) × 100) when the relative risk is greater than 1 (i.e., ‘relative risk increase’). Current CONSORT guidelines recommend reporting relative measures of association (e.g., relative risk, odds ratios, and percent relative effect) in conjunction with absolute measures of association. 

Absolute measures include the risk difference (the cumulative incidence in one group minus the cumulative incidence in the other group), rate difference (the incidence rate in one group minus the incidence rate in the other group), and the inverse of risk difference or rate difference, known as the ‘number needed to treat’ or ‘number needed to harm’ when the intervention decreases or increases risk, respectively. The number needed to treat (or harm) signifies the number of people who would require the intervention/exposure to prevent (or cause) one additional poor outcome.

## Figures and Tables

**Table 1:** The incidence of prostate cancer in men aged 50–75 years receiving daily dutasteride versus placebo during 4 years of study.

		Incident Prostate Cancer		
		Yes	No	Row Total
Dutasteride Therapy	Yes	659	2646	3305
	No	858	2566	3424
	Column Total	1517	5212	6729

Note: Results unadjusted for simplicity [22].

**Table 2:** A standard contingency table showing the possible combinations of exposure/intervention status and outcome status.

		Outcome of Interest	
		Positive	Negative
Exposure/Intervention	Present	A	B
	Absent	C	D

**Table 3:** The risk and odds of prostate cancer in men aged 50–75 years (all subjects) at varying event frequencies with no change in sample size.

Prostate Cancer	No Prostate Cancer	Row Total	Risk	Odds
Hypothetical Study Population with increased disease frequency (*n* = 6729)				
4000	2729	6729	4000/6729 = 0.59	4000/2729 = 1.47
3000	3729	6729	3000/6729 = 0.45	3000/3729 = 0.80
2000	4729	6729	2000/6729 = 0.30	2000/4729 = 0.42
Actual study population (*n* = 6729)				
1517	5212	6729	1517/6729 = 0.23	1517/5212 = 0.29
Hypothetical study population with decreased disease frequency (*n* = 6729)				
1000	5729	6729	1000/6729 = 0.15	1000/5729 = 0.17
500	6229	6729	500/6729 = 0.07	500/6229 = 0.08
100	6629	6729	100/6729 = 0.015	100/6629 = 0.015

**Table 4:** Odds ratios and relative risk of prostate cancer in men aged 50–75 years at varying event frequencies in the intervention group with no change in the sample size.

	Prostate Cancer	No Prostate Cancer	Row Total	Odds of Cancer	Odds Ratio	Risk of Cancer	Relative Risk
Hypothetical study population with increased disease frequency (*n* = 6729)							
Intervention	(↑↑↑) 2000	(↓↓↓) 1305	(--) 3305	2000/1305 = 1.53	1.53/0.33 = 4.64	2000/3305 = 0.61	0.61/0.25 = 2.44
Control	(--) 858	(--) 2566	(--) 3424	858/2566 = 0.33		858/3424 = 0.25	
Intervention	(↑↑) 1000	(↓↓) 2305	(--) 3305	1000/2305 = 0.43	0.43/0.33 = 1.30	1000/3305 = 0.30	0.30/0.25 = 1.20
Control	(--) 858	(--) 2566	(--) 3424	858/2566 = 0.33		858/3424 = 0.25	
Intervention	(↑) 828	(↓) 2477	(--) 3305	828/2477 = 0.33	0.33/0.33 = 1.0	828/3305 = 0.25	0.25/0.25 = 1.0
Control	(--) 858	(--) 2566	(--) 3424	858/2566 = 0.33		858/3424 = 0.25	
Actual study population (*n* = 6729)							
Dutasteride	659	2646	3305	659/2646 = 0.25	0.25/0.33 = 0.76	659/3305 = 0.20	0.20/0.25 = 0.8
Control	858	2566	3424	858/2566 = 0.33		858/3424 = 0.25	
Hypothetical study population with decreased disease frequency (*n* = 6729)							
Intervention	(↓) 100	(↑) 3205	(--) 3305	100/3205 = 0.03	0.03/0.33 = 0.09	100/3305 = 0.03	0.03/0.25 = 0.12
Control	(--) 858	(--) 2566	(--) 3424	858/2566 = 0.33		858/3424 = 0.25	
Intervention	(↓↓) 10	(↑↑) 3295	(--) 3305	10/3295 = 0.003	0.003/0.33 = 0.009	10/3305 = 0.003	0.003/0.25 = 0.012
Control	(--) 858	(--) 2566	(--) 3424	858/2566 = 0.33		858/3424 = 0.25	

Note: ↑/↓/-- denote an increase/decrease/no change in prostate cancer disease frequency in the hypothetical study populations relative to the actual study population, respectively.

**Table 5:** High radon exposure status in lung cancer cases versus controls.

		Primary Outcome (Lung Cancer)	
		Yes (Cases)	No (Controls)
Exposure (High Residential Radon)	Yes	192	195
	No	297	556
	Total	489	751

**Table 6:** The incidence of bladder cancer in subjects with versus without bladder diverticula in a Taiwanese population-based cohort of hospitalized urology patients.

		**Primary Outcome** **(Bladder Cancer)**			**Total Person-Years** **of Observation**
		Yes	No	Row Total	
Exposure (Bladder Diverticulum)	Yes	37	2097	2134	11,674
	No	58	8470	8528	47,711

## Data Availability

Not applicable.
